# Neoadjuvant-Adjuvant vs Neoadjuvant-Only PD-1 and PD-L1 Inhibitors for Patients With Resectable NSCLC: An Indirect Meta-Analysis

**DOI:** 10.1001/jamanetworkopen.2024.1285

**Published:** 2024-03-07

**Authors:** Yixin Zhou, Anlin Li, Hui Yu, Yuhong Wang, Xuanye Zhang, Huijuan Qiu, Wei Du, Linfeng Luo, Sha Fu, Li Zhang, Shaodong Hong

**Affiliations:** 1 State Key Laboratory of Oncology in South China, Collaborative Innovation Center for Cancer Medicine, Sun Yat-sen University Cancer Center, Guangzhou, China; 2 Department of VIP Region, Sun Yat-sen University Cancer Center, Guangzhou, China; 3 Department of Medical Oncology, Sun Yat-sen University Cancer Center, Guangzhou, China; 4 Department of Endoscopy, Sun Yat-sen University Cancer Center, Guangzhou, China; 5 Guangdong Provincial Key Laboratory of Malignant Tumor Epigenetics and Gene Regulation, Guangzhou, China; 6 Department of Cellular and Molecular Diagnostics Center, Sun Yat-sen Memorial Hospital, Sun Yat-sen University, Guangzhou, China

## Abstract

**Importance:**

Neoadjuvant therapy combining programmed cell death 1 (PD-1) and programmed death ligand 1 (PD-L1) inhibitors with platinum-based chemotherapy has demonstrated significant improvement in pathologic response and survival rates among patients with resectable non–small cell lung cancer (NSCLC). However, it remains controversial whether PD-1 blockade therapy given before and after surgery (neoadjuvant-adjuvant treatment) is associated with better outcomes than when given only before surgery (neoadjuvant-only treatment).

**Objective:**

To compare the efficacy and safety associated with neoadjuvant-adjuvant anti–PD-1 and anti–PD-L1 therapy with neoadjuvant-only anti–PD-1 and anti–PD-L1 therapy for patients with resectable NSCLC.

**Data Sources:**

A systematic search was conducted across databases including PubMed, Embase, and the Cochrane Library, as well as major oncology conferences, through July 31, 2023.

**Study Selection:**

Randomized clinical trials comparing neoadjuvant-adjuvant or neoadjuvant-only PD-1 and PD-L1 inhibitor therapy vs chemotherapy alone for patients with resectable NSCLC were selected.

**Data Extraction and Synthesis:**

Following the Preferred Reporting Items for Systematic Reviews and Meta-analyses (PRISMA) reporting guideline, 2 authors independently extracted data. Hazard ratios (HRs) and 95% CIs for event-free survival (EFS) and overall survival (OS) were extracted and then pooled through the generic inverse-variance methods. Relative risks (RRs) for treatment-related adverse events (TRAEs) were derived via the Mantel-Haenszel method. Using chemotherapy as a common comparator, indirect comparisons between neoadjuvant-adjuvant immunotherapy and neoadjuvant-only immunotherapy were conducted using frequentist methods. A random or fixed model was used based on intertrial heterogeneity identified through the Cochran *Q* test.

**Main Outcomes and Measures:**

The primary outcome was EFS, with secondary outcomes including OS and TRAEs.

**Results:**

The study encompassed 4 trials of neoadjuvant-adjuvant immunotherapy and 1 trial of neoadjuvant-only immunotherapy, involving 2385 patients. Direct meta-analysis revealed significant improvements in EFS for both neoadjuvant-adjuvant and neoadjuvant-only immunotherapy compared with chemotherapy alone. In indirect meta-analysis, the addition of adjuvant immunotherapy to neoadjuvant immunotherapy was not associated with improved EFS (HR, 0.90; 95% CI, 0.63-1.30; *P* = .59) or OS (HR, 1.18; 95% CI, 0.73-1.90; *P* = .51) compared with neoadjuvant-only immunotherapy. Moreover, the incidence of any grade of TRAEs significantly increased with the addition of adjuvant immunotherapy (RR, 1.08; 95% CI, 1.00-1.17; *P* = .04).

**Conclusions and Relevance:**

This meta-analysis suggests that adding PD-1 or PD-L1 inhibitors in the adjuvant phase to neoadjuvant treatment with PD-1 or PD-L1 inhibitors and chemotherapy may not improve survival outcomes for patients with resectable NSCLC and may be associated with increased adverse events. Future validation of these findings is warranted through head-to-head randomized clinical trials.

## Key Points

**Question:**

Is administration of programmed cell death 1 (PD-1) and programmed death ligand 1 (PD-L1) inhibitors both before and after surgery (neoadjuvant-adjuvant treatment) associated with better outcomes than administration of the therapy solely before surgery (neoadjuvant-only treatment) in patients with resectable non–small cell lung cancer (NSCLC)?

**Findings:**

In this meta-analysis, the addition of PD-1 and PD-L1 inhibitors in the adjuvant phase, alongside neoadjuvant PD-1 and PD-L1 inhibitors and chemotherapy, was not associated with a significant improvement in event-free survival or overall survival for resectable NSCLC. Neoadjuvant-adjuvant treatment was also associated with an increase in treatment-related adverse events.

**Meaning:**

This study suggests that adding adjuvant immunotherapy may not improve clinical outcomes for patients with early-stage NSCLC who receive neoadjuvant PD-1 and PD-L1 inhibitors plus chemotherapy and undergo radical surgery.

## Teaser

This meta-analysis compares the efficacy and safety of neoadjuvant-adjuvant anti–programmed cell death 1 (PD-1) and anti–programmed death ligand 1 (PD-L1) therapy with neoadjuvant-only anti–PD-1 and anti–PD-L1 therapy for patients with resectable non–small cell lung cancer (NSCLC).

## Introduction

Approximately 20% to 30% of patients with non–small cell lung cancer (NSCLC) initially present with resectable disease^1^; however, 30% to 55% develop recurrence and die of the disease despite surgical excision.^2^ Given the high risk of relapse, multidisciplinary research has been the academic focus for decades. Despite use of platinum-based neoadjuvant or adjuvant chemotherapy, both approaches showed comparable yet marginal improvement in overall survival (OS).^3,4^ Recently, the landscape of adjuvant and neoadjuvant systemic therapy has evolved, incorporating immune checkpoint inhibitors and targeted therapies with the approval of 4 regimens (nivolumab, atezolizumab, pembrolizumab, and osimertinib) based on clinical trials including CheckMate 816, IMpower010, PEARLS/KEYNOTE-091, and ADAURA.^5,6,7,8^ Different treatment scenarios are now available for resectable NSCLC, leading to debates about the optimal choices.

Perioperative immunotherapy holds great promise in resectable NSCLC due to reduced tumor clonal heterogeneity and enhanced fitness of host immunity in earlier-stage tumors.^9,10^ In the neoadjuvant setting, the primary tumor could serve as a neoantigen source for priming, expansion, and activation of tumor-specific T cells. In addition, the intact interaction between the primary tumor and the uninvolved tumor-draining lymph nodes is essential for antitumor immune response, favoring a neoadjuvant immunotherapy strategy.^11,12^ Preclinical and clinical work further suggests that neoadjuvant administration of immune checkpoint inhibitors may be superior to adjuvant therapy.^13,14^ The CheckMate 816 trial, in which patients with resectable NSCLC received neoadjuvant chemotherapy plus nivolumab or chemotherapy alone before undergoing definitive surgery, demonstrated significant improvement in the pathologic complete response (pCR) rate (24.0% vs 2.2%) and event-free survival (EFS; hazard ratio [HR], 0.63 [95% CI, 0.43-0.91]).^5^ Subsequently, randomized studies investigating anti–programmed cell death 1 (anti–PD-1) and anti–programmed death ligand 1 (anti–PD-L1) neoadjuvant therapy for patients with resectable NSCLC have emerged, including the KEYNOTE-671 trial with pembrolizumab,^15^ the Neotorch trial with toripalimab,^16^ the AEGEAN trial with durvalumab,^17^ and the NADIM II trial with nivolumab.^18^ These trials built on the neoadjuvant treatment strategy of CheckMate 816; however, they have introduced additional adjuvant immunotherapy, which was waived in the CheckMate 816 study. This neoadjuvant-adjuvant approach is attractive because the IMpower010^6^ and KEYNOTE-091^7^ trials demonstrated that adjuvant-only immunotherapy extended the disease-free survival of patients with surgically resected NSCLC. Furthermore, the potential presence of micrometastases after the CheckMate 816 strategy may be associated with disease relapse, which may need to be counteracted with additional postoperative treatment. Therefore, there is a need to test whether administration of PD-1 and PD-L1 inhibitor therapy before and after surgery (neoadjuvant-adjuvant treatment) would result in better outcomes than administration only before surgery (neoadjuvant-only treatment) among patients with resectable NSCLC.

Considering that a randomized clinical trial to specifically address this issue is unlikely to be available in the near future, we conducted this meta-analysis by pooling data from pivotal neoadjuvant immunotherapy trials involving more than 2000 patients to compare the benefits and toxic effects of neoadjuvant-adjuvant anti–PD-1 and anti–PD-L1 therapy with neoadjuvant-only treatment in resectable NSCLC.

## Method

This meta-analysis followed the Preferred Reporting Items for Systematic Reviews and Meta-analyses (PRISMA) reporting guideline.^19^ The study was registered on the International Platform of Registered Systematic Review and Meta-analysis Protocols (INPLASY; 2023120074). An exemption from the institutional review board of Sun Yat-sen University Cancer Center was obtained based on the nonharmful nature of the study.

### Data Sources and Search Strategy

A comprehensive literature search was conducted across databases including PubMed, Embase, and the Cochrane Library, supplemented by investigations from prominent oncology conferences, from database inception to July 31, 2023. Search parameters encompassed primary terms as *PD-1/PD-L1*, *adjuvant/neoadjuvant/perioperative*, *non–small cell lung carcinoma*, and *randomized controlled trials* (details provided in eMethods in Supplement 1).

### Inclusion Criteria

Included studies (1) focused on patients with resectable NSCLC; (2) involved PD-1 or PD-L1 inhibitors in neoadjuvant or adjuvant therapy; (3) compared groups receiving immunotherapy with control groups receiving chemotherapy alone; (4) reported EFS, OS, and/or treatment-related adverse events (TRAEs); and (5) were designed to be randomized clinical prospective studies.

### Data Extraction and Assessment of Study Quality

Two authors (Y.Z. and A.L.) independently extracted data and resolved discrepancies by consensus. Collected data pertained to outcomes including EFS and its subgroup data, OS, and TRAEs. Ancillary details were recorded in the predefined information sheet. Methodological integrity was assessed using the Cochrane Risk of Bias Tool.^20^

### Main Outcomes

The primary outcome was EFS, defined as the duration from randomization to either local progression impeding planned surgery, an unresectable tumor, disease progression or recurrence, or death. The secondary outcomes included OS and TRAEs. Regarding TRAEs, our focus was on TRAEs of any grade, grades 3 to 5, leading to discontinuation, serious adverse events, and those resulting in death.

### Statistical Analysis

The analysis encompassed 3 distinct treatment strategies: (1) neoadjuvant anti–PD-1 or anti–PD-L1 therapy plus chemotherapy followed by surgical resection and subsequent treatment with an adjuvant anti–PD-1 or anti–PD-L1 inhibitor, henceforth referred to as the neoadjuvant-adjuvant immunotherapy group (arm A); (2) neoadjuvant PD-1 or PD-L1 inhibitor plus chemotherapy, devoid of adjuvant immunotherapy, designated as the neoadjuvant-only immunotherapy group (arm B); and (3) neoadjuvant chemotherapy alone, which served as the control group (arm C).

Initially, we performed direct meta-analysis comparing neoadjuvant-adjuvant and neoadjuvant-only immunotherapy with chemotherapy alone. The pooled HRs for both EFS and OS were determined through the generic inverse-variance methods model, whereas pooled relative risks (RRs) for TRAEs were derived via the Mantel-Haenszel method.^21^ Intertrial heterogeneity was examined through the Cochran *Q* test, with *P* < .10 and *I*^2^ > 50% demarcating significant heterogeneity—in such instances, a random-effects model was used; otherwise, a fixed-effects model was applied.^22^ Publication bias was assessed by scrutinizing the funnel plot of each trial’s effect size against its reciprocal SE, bolstered by the Egger test.^22^

Using arm C as a fulcrum, an indirect comparison between arm A and arm B was conducted using frequentist methods with the following formula^23^: log HR_AB_ = log HR_AC_ − log HR_BC_, and its SE for the log HR was SE (log HR_AB_) = [SE (log HR_AC_)^2^ + SE (log HR_BC_)^2^]^1/2^. Relative risk was analogously computed. For the subgroup analysis, we extracted relevant data from each study’s subgroups, pooled them through direct meta-analysis, and then applied the same indirect comparison formula as used in the overall analysis. To assess heterogeneity in treatment effects on EFS across subgroups, we conducted a meta-regression using the restricted maximum likelihood method.^24,25^ The interaction term’s *P* values for subgroups were derived through this analysis, using the metafor package in R, version 4.2.1 (R Project for Statistical Computing).

Other statistical analyses were conducted with Stata software, version 16.0 (StataCorp). All tests were 2-tailed, and *P* < .05 was deemed statistically significant.

## Results

**Figure 1.**
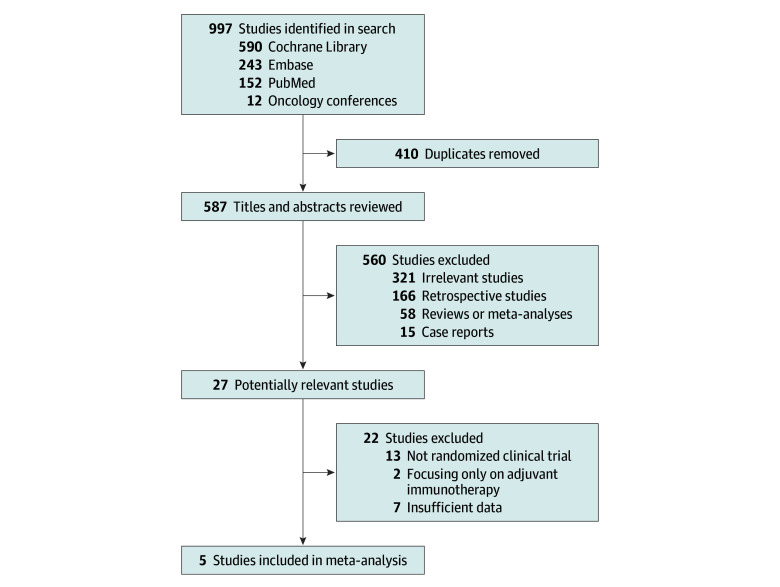
Study Selection Studies selected based on the Preferred Reporting Items for Systematic Reviews and Meta-analyses (PRISMA) reporting guideline.

A total of 5 trials, including 2385 patients, were analyzed (detailed flowchart in Figure 1). Details of the bias assessment are provided in eTable 1 in Supplement 1. A primary source of bias arose from the Neotorch trial because data were obtained from a conference proceeding.

### Trial Characteristics and Outcomes

The characteristics and outcomes of the included trials are presented in Table 1.^5,15,16,17,18^ Four trials (KEYNOTE-671, Neotorch, AEGEAN, and NADIM II) explored neoadjuvant-adjuvant immunotherapy vs chemotherapy alone, while 1 trial (CheckMate 816) investigated neoadjuvant-only immunotherapy. All included trials involved patients with a diagnosis of stage II to III resectable tumors (per the American Joint Commission on Cancer Staging Manual 8th edition guidelines^26^) except for NADIM II, which focused exclusively on patients with stage III tumors. In all experimental groups across these trials, 3 to 4 cycles of neoadjuvant PD-1 or PD-L1 inhibitor plus platinum-based chemotherapy were administered. Predominantly, patients received a year of adjuvant immunotherapy, except in NADIM II (6 months). With a median follow-up time ranging from 7.8 to 23.9 months, none of the trials could pinpoint a median EFS for the experimental groups. Conversely, a median EFS of 15.5 to 25.9 months was observed in the control arms. Across all studies, 2-year survival rates were consistently elevated in the experimental arm vs the control, with similar data observed between the neoadjuvant-adjuvant immunotherapy trials (80.9%-85.0%) and the neoadjuvant-only immunotherapy trial (82.7%). Additional characteristics of the enrolled trials are available in eTable 2 in Supplement 1.

**Table 1.** Characteristics of Patients and Outcomes of Included Trials

Trial name	Adjuvant immunotherapy duration, y	Stage^a^	Primary end point	No.	Arm	Neoadjuvant cycles	Median follow-up, mo	Median EFS, mo	HR for EFS	2-y OS, %	HR for OS
CheckMate 816,^5^ 2022	Neoadjuvant only	II-III^b^	EFS and pCR	179	Chemotherapy	3	41.4	21.1	1 [Reference]	70.6	1 [Reference]
				179	Nivolumab + chemotherapy			NR	0.63 (0.45-0.87)	82.7	0.57 (0.30-1.07)
KEYNOTE-671,^15^ 2023	1	II-III	EFS and OS	400	Chemotherapy	4	25.2	17.0	1 [Reference]	77.6	1 [Reference]
				397	Pembrolizumab + chemotherapy			NR	0.58 (0.46-0.72)	80.9	0.73 (0.54-0.99)
Neotorch,^16^ 2023	1	II-III	EFS and MPR	202	Chemotherapy	3	18.3	15.5	1 [Reference]	74.3	1 [Reference]
				202	Toripalimab + chemotherapy			NR	0.40 (0.27-0.57)	81.2	0.62 (0.38-1.00)
AEGEAN,^17^ 2023	1	II-III	EFS and pCR	374	Chemotherapy	4	11.7	25.9	1 [Reference]	NR	1 [Reference]
				366	Durvalumab + chemotherapy			NR	0.68 (0.53-0.88)	NR	NR
NADIM II,^18^ 2023	0.5	III	pCR	29	Chemotherapy	3	26.1	NR	1 [Reference]	63.6	1 [Reference]
				57	Nivolumab + chemotherapy			NR	0.47 (0.25-0.88)	85.0	0.43 (0.19-0.98)

Abbreviations: EFS, event-free survival; HR, hazard ratio; MPR, major pathologic response; NR, not reported; OS, overall survival; pCR, pathologic complete response.

^a^ According to the 8th edition of the American Joint Commission on Cancer Staging Manual.

^b^ The trial enrolled patients with stage IB (tumors ≥4 cm) according to the 7th edition of the American Joint Commission on Cancer Staging Manual, which was reclassified as stage IIA according to the 8th edition.

### Direct Meta-Analysis for EFS

**Figure 2.**
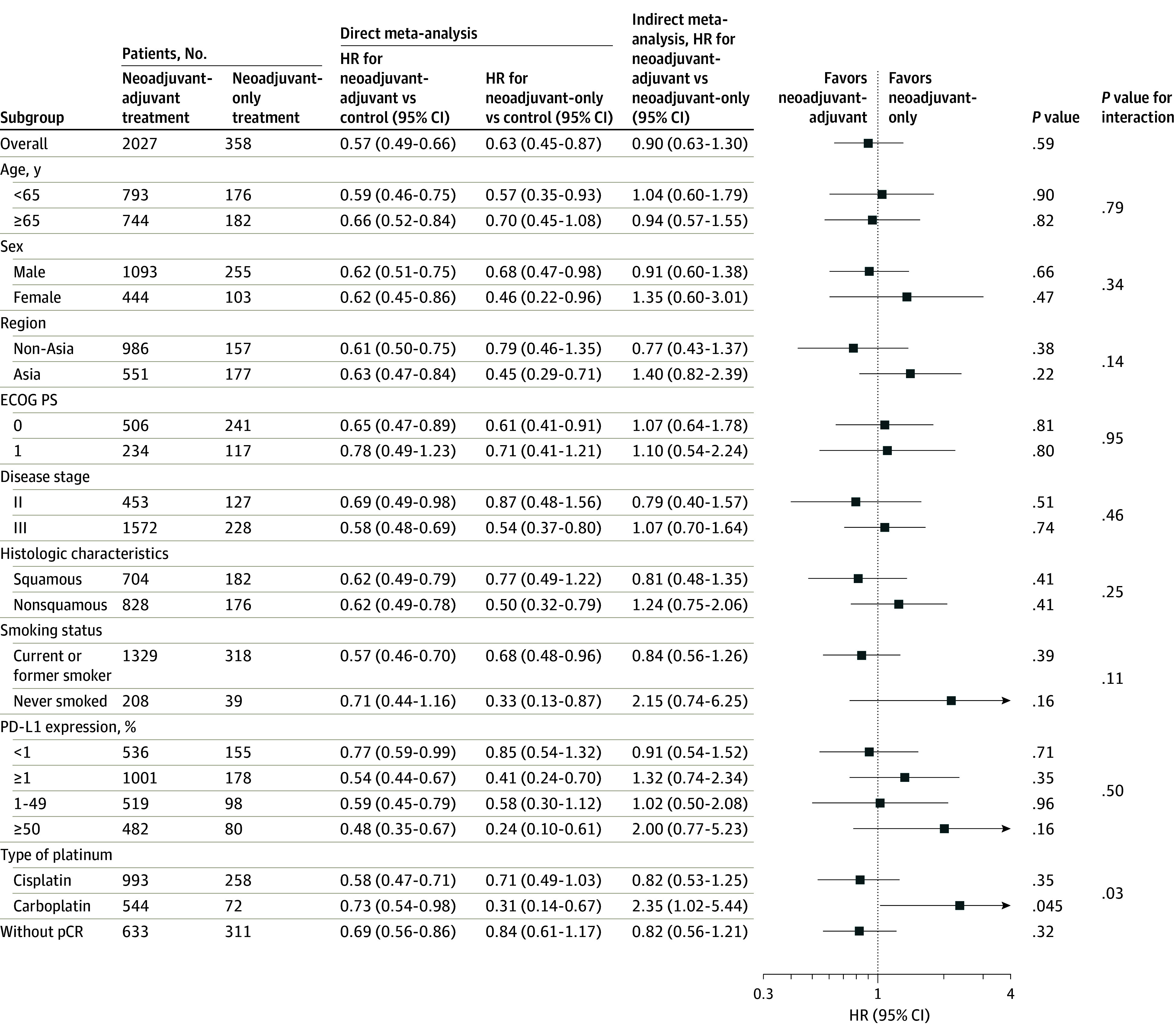
Overall and Subgroup Analysis of Event-Free Survival (EFS) Between Neoadjuvant-Adjuvant and Neoadjuvant Programmed Cell Death 1 (PD-1) and Programmed Death Ligand 1 (PD-L1) Inhibitors for Completely Resected Non–Small Cell Lung Cancer (NSCLC) The fourth column contains data summarizing the direct meta-analysis results for EFS when comparing neoadjuvant-adjuvant PD-1 or PD-L1 inhibitors with the control group (neoadjuvant chemotherapy only). Fixed-effect models were applied due to the low heterogeneities. The original forest plots are available in eFigure 1 in Supplement 1. In the fifth column, the summarized outcomes of neoadjuvant-only PD-1 or PD-L1 inhibitors vs the control group are presented. The sixth and seventh columns display the forest plot of hazard ratios (HRs) indirectly comparing EFS between neoadjuvant-adjuvant and neoadjuvant-only anti–PD-1 and anti–PD-L1 therapy, with the associated *P* value summarized in the eighth column. The *P* value for interaction in the ninth column indicates the significance of the difference between subgroups. All statistical tests were conducted with a 2-sided approach. ECOG indicates Eastern Cooperative Oncology Group; pCR, pathologic complete response; and PS, performance status.

We began our analyses with a direct meta-analysis to determine the pooled HR for EFS comparing neoadjuvant-adjuvant immunotherapy with chemotherapy alone, both for the entire cohort and for specific predefined subgroups (eFigure 1 in Supplement 1). Fixed-effects models were applied due to the low heterogeneities. The data are summarized in Figure 2.

A significant difference was found in EFS favoring neoadjuvant-adjuvant immunotherapy vs chemotherapy alone (HR, 0.57; 95% CI, 0.49-.66; *P* < .001; *I^2^* = 47.3%) (Figure 2). The funnel plot revealed no asymmetry (eFigure 2 in Supplement 1; Egger test *P* = .38). This benefit was seen in all subgroups. However, 2 specific subgroups did not reach statistical significance: patients with an Eastern Cooperative Oncology Group (ECOG) performance status of 1 (HR, 0.78; 95% CI, 0.49-1.23) and never smokers (HR, 0.71; 95% CI, 0.44-1.16) (Figure 2). The pooled HR for those with PD-L1 expression of 50% or more was 0.48 (95% CI, 0.35-0.67). In the comparison of neoadjuvant-only immunotherapy with chemotherapy alone, the HR for EFS was 0.63 (95% CI, 0.45-0.87). Subgroup results are presented in Figure 2.

### Indirect Meta-Analysis for EFS

Indirect meta-analysis comparing neoadjuvant-adjuvant immunotherapy with neoadjuvant-only immunotherapy is also presented in Figure 2. The 2 strategies were associated with comparable treatment efficacy in terms of EFS (HR, 0.90; 95% CI, 0.63-1.30; *P* = .59). This similarity prevailed across most subgroups, including age, gender, region, ECOG performance status, disease stage, and histologic characteristics. For those not achieving pCR from neoadjuvant therapy, findings indicate a numerically improved EFS (nonsignificant) with additional adjuvant therapy (HR, 0.82; 95% CI, 0.56-1.21; *P* = .32). Conversely, specific subgroups, such as never smokers and those with PD-L1 expression of 50% or more, showed a numerically inferior (nonsignificant) EFS outcome. Patients receiving a carboplatin-based chemotherapy regimen demonstrated a significantly inferior EFS outcome with adjuvant immunotherapy (HR, 2.35; 95% CI, 1.02-5.44; *P* = .045). The interaction term’s *P* value between carboplatin-based and cisplatin-based regimens was *P* = .03.

### Meta-Analysis Results for OS

Regarding OS, both neoadjuvant-adjuvant and neoadjuvant-only immunotherapies were associated with significantly reduced risk of death compared with chemotherapy alone (HR for neoadjuvant-adjuvant immunotherapy vs control, 0.67; 95% CI, 0.52-0.85; *I^2^* = 0.0%; eFigure 3 in Supplement 1; Egger test *P* = .04; HR for neoadjuvant-only immunotherapy vs control, 0.57; 95% CI, 0.38-0.87). Indirect analysis further revealed no significant difference in OS between the 2 regimens (HR for neoadjuvant-adjuvant immunotherapy vs control, 1.18; 95% CI, 0.73-1.90; *P* = .51).

### Safety

The results of analyses for safety are summarized in Table 2. In the neoadjuvant-adjuvant immunotherapy group, the incidences of any grade of TRAEs and grade 3 to 5 TRAEs were 86.5% (346 of 400) to 99.5% (201 of 202) and 32.3% (129 of 400) to 63.4% (128 of 202), respectively, in the included trials. Through direct meta-analysis, we found that compared with chemotherapy alone, the addition of neoadjuvant-adjuvant immunotherapy was associated with a numerically higher (nonsignificant) incidence of any grade TRAEs (RR, 1.02; 95% CI, 1.00-1.04; *P* = .09), significantly increased the incidence of grade 3 or above TRAEs (RR, 1.14; 95% CI, 1.03-1.26; *P* = .01), was associated with more treatment discontinuation (RR, 1.92; 95% CI, 1.38-2.68; *P* < .001), and resulted in more serious adverse events (RR, 1.26; 95% CI, 1.09-1.45; *P* = .001) (forest plots shown in eFigure 4 in Supplement 1). In contrast, the addition of immunotherapy in the neoadjuvant-only setting did not significantly increase the incidence of TRAEs of any grade (RR, 0.94; 95% CI, 0.87-1.01) or grade 3 or above TRAEs (RR, 0.93; 95% CI, 0.71-1.22) (Table 2; eFigure 5 in Supplement 1). In indirect analysis comparing neoadjuvant-adjuvant and neoadjuvant immunotherapies, the addition of adjuvant immunotherapy significantly increased the incidence of TRAEs of any grade (RR, 1.08; 95% CI, 1.00-1.17; *P* = .04), and data indicate a nonsignificantly higher incidence of grade 3 to 5 TRAEs (RR, 1.23; 95% CI, 0.92-1.64; *P* = .17) and TRAEs leading to treatment discontinuation (RR, 1.52; 95% CI, 0.84-2.76; *P* = .17) (Table 2). The KEYNOTE-671 and NADIM II studies reported a 54.5% (158 of 290) and 56.8% (25 of 44) incidence of TRAEs of any grade, respectively, and 10.0% (29 of 290) and 4.5% (2 of 22) incidence of grade 3 to 5 TRAEs, respectively, in the adjuvant immunotherapy phase. Moreover, the incidence of TRAEs leading to death was between 1.0% (4 of 396) and 3.0% (6 of 202) in the neoadjuvant-adjuvant immunotherapy group, whereas no treatment-related fatal events were reported in the neoadjuvant-only immunotherapy group.

**Table 2.** Comparisons of RRs Regarding TRAEs Among Different Treatment Regimens

Event	TRAEs, No. (%)					RR (95% CI)			*P* value	TRAEs in adjuvant phase, No. (%)	
	KEYNOTE-671 (n = 396)	Neotorch (n = 202)	AEGEAN (n = 400)	NADIM II (n = 57)^a^	CheckMate 816 (n = 176)	Neoadjuvant-adjuvant vs control^b^	Neoadjuvant vs control^c^	Neoadjuvant-adjuvant vs neoadjuvant		KEYNOTE-671 (n = 290)	NADIM II (n = 44)
Any grade	383 (96.7)	201 (99.5)	346 (86.5)	50 (87.7)	145 (82.4)	1.02 (1.00-1.04)	0.94 (0.87-1.01)	1.08 (1.00-1.17)	.04	158 (54.5)	25 (56.8)
Grades 3-5	178 (44.9)	128 (63.4)	129 (32.3)	12 (21.1)	59 (33.5)	1.14 (1.03-1.26)	0.93 (0.71-1.22)	1.23 (0.92-1.64)	.17	29 (10.0)	2 (4.5)
Led to discontinuation	50 (12.6)	19 (9.4)	48 (12.0)	4 (7.0)	28 (15.9)	1.92 (1.38-2.68)	1.26 (0.77-2.07)	1.52 (0.84-2.76)	.17	NR	NR
Serious AEs	70 (17.7)	82 (40.6)	150 (37.5)	NR	36 (20.5)	1.26 (1.09-1.45)	1.13 (0.73-1.73)	1.12 (0.71-1.76)	.64	16 (5.5)	NR
Led to death	4 (1.0)	6 (3.0)	7 (1.8)	0	0	NA	NA	NA	NA	1 (0.3)	0

Abbreviations: AEs, adverse events; NA, not available to access due to insufficient data; NR, not reported; RR, relative risk; TRAEs, treatment-related adverse events.

^a^ The present data were TRAEs during the neoadjuvant phase.

^b^ The KEYNOTE-671, Neotorch, and AEGEAN study data were pooled. The forest plot is available in eFigure 4 in Supplement 1.

^c^ Because data regarding TRAEs in the NADIM II study were collected during the neoadjuvant phase, the data were pooled with data from the NADIM II and CheckMate 816 trials. The forest plot is in eFigure 5 in Supplement 1.

## Discussion

This indirect meta-analysis compared the efficacy and safety of neoadjuvant-adjuvant anti–PD-1 and anti–PD-L1 treatment with neoadjuvant-only anti–PD-1 and anti–PD-L1 treatment for patients with resectable NSCLC. Results revealed that adding immunotherapy in the adjuvant phase to neoadjuvant immunotherapy plus chemotherapy was not associated with a significant improvement in EFS or OS vs neoadjuvant immunotherapy plus chemotherapy alone. However, it did increase incidence of TRAEs. Our analysis indicates that neoadjuvant immunotherapy plus platinum-based chemotherapy might be a preferable treatment for patients with resectable NSCLC, sparing them from adjuvant immunotherapy, which increases financial burden and toxic effects while not improving survival outcomes.

Our results imply that the timing of immune checkpoint inhibitor administration related to radical surgery is critical for patients’ outcomes and provide a basis to reconsider the role of adjuvant immunotherapy for patients with resected cancers. The findings further support the theory that immunotherapy given in the neoadjuvant phase could elicit a more enhanced systemic antitumor response when neoantigen-bearing tumor and antitumor T cells are not yet surgically resected. The shared effects of eliminating micrometastatic foci through immunotherapy, whether administered before or after surgery, may at least partially explain the lack of benefit with additional adjuvant immunotherapy. Adjuvant-only immunotherapy also has been approved for patients with completely resected NSCLC based on the IMpower010 and PEARLS/KEYNOTE-091 trials. However, there is no available randomized clinical trial to specifically compare neoadjuvant-only immunotherapy with adjuvant-only immunotherapy for patients with lung cancer. Preclinical work with breast cancer murine models points out that neoadjuvant immunotherapy exhibits greater efficacy than the adjuvant approach.^13^ Two clinical trials also show that moving 2 to 3 courses of immunotherapy to the neoadjuvant phase outperforms giving immunotherapy to patients solely in the adjuvant setting.^14,27^

Although our current results discourage adjuvant immunotherapy for unselected patients who have received neoadjuvant immunotherapy plus chemotherapy, it becomes a renewed unmet need to develop novel strategies to reinforce the efficacy of neoadjuvant-only immunotherapy as well as to find out which patient population would derive benefits from additional adjuvant immunotherapy. To achieve this goal, high-dimensional multiomics investigations within the neoadjuvant treatment framework would be necessary.^28^ In the present study, we sought to address this issue in a preliminary manner through subgroup analysis. Our analysis indicated that patients treated with carboplatin exhibited an inferior EFS with additional adjuvant immunotherapy. This observation primarily stems from the notably low HR (0.31 [95% CI, 0.14-0.67]) reported in this subgroup within the CheckMate 816 study. A plausible explanation offered in the study is the relatively small sample size in this subgroup, which might have resulted in the analyses lacking sufficient statistical power.^5^ None of the other subgroups exhibited differential EFS outcomes between neoadjuvant-adjuvant and neoadjuvant-only approaches. Nonetheless, some signals are worth noting. In the CheckMate 816 trial, patients achieving tumor pCR experienced a 2-year EFS rate exceeding 90%, contrasting with approximately 50% among those without pCR (HR, 0.13; 95% CI, 0.05-0.37). This finding provides an attractive basis to investigate whether the extent of pathologic response observed at surgery could guide adjuvant treatment escalation or deescalation strategies. In our comparative analysis between neoadjuvant-adjuvant and neoadjuvant-only groups in the non-pCR subgroup, there was some evidence suggesting improved EFS with additional adjuvant immunotherapy (HR in the non-pCR subgroup, 0.82; 95% CI, 0.56-1.21). However, this result lacks statistical significance, and the magnitude of the EFS benefit remains modest. For patients with a tumor pCR, the sample size is too small (only 4 patients having a pCR in the chemotherapy arm of the CheckMate 816 trial) to conduct the aforementioned subgroup analysis. Due to the obviously low risk of disease recurrence in patients with a pCR after CheckMate 816 treatment, it is unlikely that this patient subgroup would have gained extra benefit from adjuvant immunotherapy. Rather, there is a need for clinical trials that formally assess the continuation of adjuvant immunotherapy vs observation for patients who can not attain a pCR after neoadjuvant chemoimmunotherapy. Another relevant surrogate end point affecting postsurgery treatment is circulating tumor DNA clearance or a negative molecular residual disease test result. Further insights into personalized postsurgery treatment options may stem from the elucidation of the primary factors associated with residual disease after immunotherapy through well-designed multiomics studies.

On October 10, 2023, the KEYNOTE-671 study reported achieving the end point of OS improvement, marking it as the first approach to enhance OS. Given the incomplete maturity of OS data across most trials and the potential publication bias indicated by the Egger test, it is prudent to interpret the OS results with caution. Thus, more trials and extended follow-up are needed to discern any differences in OS improvements between neoadjuvant-adjuvant and neoadjuvant-only immunotherapy approaches.

Regarding TRAEs, while individual neoadjuvant-adjuvant immunotherapy trials reported no significant increase when adding anti–PD-1 or anti–PD-L1 antibodies compared with chemotherapy alone, our pooled results revealed a 14% increase in the incidence of grade 3 and above TRAEs. Moreover, neoadjuvant-adjuvant immunotherapy increased TRAEs of all grades by 8% compared with neoadjuvant-only therapy. This finding suggests that extended exposure to anti–PD-1 or anti–PD-L1 antibodies may elevate the risk of toxic effects. Previous studies have supported this viewpoint. For instance, Owen et al^29^ found that delayed TRAEs predominantly (74%) arose in patients still undergoing anti–PD-1 treatment. KEYNOTE-671 in its adjuvant phase reported occurrence rates of 54.5% for TRAEs of all grades and 10.0% for grade 3 and above, which are relatively high. Nevertheless, the incidence of TRAEs during the adjuvant phase might be overestimated due to the nocebo effect and delayed immune-related adverse events stemming from the neoadjuvant phase. Therefore, randomized clinical studies are imperative to discern the added toxic effects of continued immunotherapy in the adjuvant phase. In addition to the risk of toxic effects, the economic burden owing to regular clinic visits for intravenous infusions of immunotherapy also should be taken into consideration. Therefore, rooted in the findings of this study, we might be inclined not to use adjuvant anti–PD-1 or anti–PD-L1 therapy for unselected patients who have previously undergone neoadjuvant immunotherapy plus chemotherapy, which is in line with current guidelines and practice paradigm.

### Limitations

This study had several limitations that render any recommendations tentative. First, the conclusions are drawn from indirect comparisons, which may not be as robust as direct comparisons. Second, the analysis was constrained by having only 1 trial on neoadjuvant-only therapy. Third, heterogeneities between different studies should be cautiously noted. In addition, the sample sizes in certain subgroups were small. Fourth, our conclusions may be affected if more studies, including the single-arm studies, were included. Upcoming prospective trials comparing neoadjuvant-adjuvant and neoadjuvant-only anti–PD-1 and anti–PD-L1 therapies are essential to corroborate these findings. Prior to that, when making clinical decisions, considerations must encompass the patient’s molecular biomarkers, tolerability to prior therapy, preferences regarding care goals, and susceptibility to autoimmune toxic effects.

## Conclusions

To our knowledge, our meta-analysis is the first to demonstrate that the addition of adjuvant anti–PD-1 or anti–PD-L1 therapy to neoadjuvant chemoimmunotherapy is not associated with improved EFS and OS for patients with resectable NSCLC. Furthermore, this approach was associated with significantly higher TRAEs. This finding suggests that the continuation of immunotherapy in the adjuvant phase may be unnecessary and that a neoadjuvant-only approach could offer comparable outcomes with fewer toxic effects. These preliminary findings can inform clinical decisions and future research directions but should be further validated in head-to-head randomized clinical trials.

## Notes

Supplement 1.
eMethods.
**eTable 1.** Quality Assessment by Cochrane Collaboration’s Tool **eTable 2.** Additional Characteristics of Patients and Outcomes of Included Trials **eFigure 1.** Forest Plot of Hazard Ratios in Subgroup-Analyses Comparing EFS in Patients Who Received Neoadjuvant-Adjuvant Anti-PD-(L)1 Therapy Versus Chemotherapy Alone in Completely Resected NSCLC **eFigure 2.** Funnel Plot Comparing Hazard Ratios Between Neoadjuvant-Adjuvant Anti-PD-(L)1 Therapy Over Chemotherapy Alone for EFS **eFigure 3.** Forest Plot of Hazard Ratios Comparing OS in Patients Who Received Neoadjuvant-Adjuvant Anti-PD-(L)1 Therapy Versus Chemotherapy Alone in Completely Resected NSCLC **eFigure 4.** Forest Plot of Risk Ratios Comparing TRAES in Patients Who Received Neoadjuvant-Adjuvant Anti-PD-(L)1 Therapy Versus Chemotherapy Alone in Completely Resected NSCLC **eFigure 5.** Forest Plot of Risk Ratios Comparing TRAES in Patients Who Received Neoadjuvant-Only Anti-PD-(L)1 Therapy Versus Chemotherapy Alone in Completely Resected NSCLC

Supplement 2.
Data Sharing Statement
