# Supplementary material for: Neoadjuvant-Adjuvant vs Neoadjuvant-Only PD-1 and PD-L1 Inhibitors for Patients With Resectable NSCLC: An Indirect Meta-Analysis
Source: JAMA Netw Open. 2024 Mar 7;7(3):e241285. doi: 10.1001/jamanetworkopen.2024.1285 (PMC10921251; doi:10.1001/jamanetworkopen.2024.1285)
Supplement: Supplement 1. — eMethods. eTable 1. Quality Assessment by Cochrane Collaboration’s Tool eTable 2. Additional Characteristics of Patients and Outcomes of Included Trials eFigure 1. Forest Plot of Hazard Ratios in Subgroup-Analyses Comparing EFS in Patients Who Received Neoadjuvant-Adjuvant Anti-PD-(L)1 Therapy Versus Chemotherapy Alone in Completely Resected NSCLC eFigure 2. Funnel Plot Comparing Hazard Ratios Between Neoadjuvant-Adjuvant Anti-PD-(L)1 Therapy Over Chemotherapy Alone for EFS eFigure 3. Forest Plot of Hazard Ratios Comparing OS in Patients Who Received Neoadjuvant-Adjuvant Anti-PD-(L)1 Therapy Versus Chemotherapy Alone in Completely Resected NSCLC eFigure 4. Forest Plot of Risk Ratios Comparing TRAES in Patients Who Received Neoadjuvant-Adjuvant Anti-PD-(L)1 Therapy Versus Chemotherapy Alone in Completely Resected NSCLC eFigure 5. Forest Plot of Risk Ratios Comparing TRAES in Patients Who Received Neoadjuvant-Only Anti-PD-(L)1 Therapy Versus Chemotherapy Alone in Completely Resected NSCLC [file jamanetwopen-e241285-s001.pdf]

Supplementary Online Content

Zhou Y, Li A, Yu H, et al. Neoadjuvant-adjuvant vs neoadjuvant-only PD-1 and PD-L1 inhibitors for patients with resectable NSCLC: an indirect meta-analysis. *JAMA Netw Open.* 2024;7(3):e241285. doi:10.1001/jamanetworkopen.2024.1285

eMethods.
eTable 1. Quality Assessment by Cochrane Collaboration's Tool
eTable 2. Additional Characteristics of Patients and Outcomes of Included Trials
eFigure 1. Forest Plot of Hazard Ratios in Subgroup-Analyses Comparing EFS in Patients Who Received Neoadjuvant-Adjuvant Anti-PD-(L)1 Therapy Versus Chemotherapy Alone in Completely Resected NSCLC
eFigure 2. Funnel Plot Comparing Hazard Ratios Between Neoadjuvant- Adjuvant Anti-PD-(L)1 Therapy Over Chemotherapy Alone for EFS
eFigure 3. Forest Plot of Hazard Ratios Comparing OS in Patients Who Received Neoadjuvant- Adjuvant Anti-PD-(L)1 Therapy Versus Chemotherapy Alone in Completely Resected NSCLC
eFigure 4. Forest Plot of Risk Ratios Comparing TRAES in Patients Who Received Neoadjuvant-Adjuvant Anti-PD-(L)1 Therapy Versus Chemotherapy Alone in Completely Resected NSCLC
eFigure 5. Forest Plot of Risk Ratios Comparing TRAES in Patients Who Received Neoadjuvant-Only Anti-PD-(L)1 Therapy Versus Chemotherapy Alone in Completely Resected NSCLC

This supplementary material has been provided by the authors to give readers additional information about their work.

© 2024 Zhou Y et al. *JAMA Network Open.*

eMethods.

Search strategies for PubMed, EMBASE, and Cochrane database

Pubmed:152 results

(((("pembrolizumab" [Supplementary Concept] OR "lambrolizumab" [Title/Abstract] OR "Keytruda" [Title/Abstract] OR "MK-3475" [Title/Abstract] OR "nivolumab" [Supplementary Concept] OR "MDX-1106" [Title/Abstract] OR "ONO-4538" [Title/Abstract] OR "BMS-936558" [Title/Abstract] OR "Opdivo"[Title/Abstract] OR "atezolizumab"[Supplementary Concept] OR "MPDL3280A"[Title/Abstract] OR "Tecentriq"[Title/Abstract] OR"RG7446"[Title/Abstract] OR "RG-7446"[Title/Abstract] OR "Durvalumab" [Title/Abstract] OR "Imfinzi" [Title/Abstract] OR " MEDI4736" [Title/Abstract] OR "Camrelizumab" [Title/Abstract] OR "SHR-1210" [Title/Abstract] OR " Tislelizumab" [Title/Abstract] OR "Sintilimab" [Title/Abstract] OR " IBI 308" [Title/Abstract] OR "anti-PDL1"[Title/Abstract] OR "anti-PD1"[Title/Abstract] OR "PD-1"[Title/Abstract] OR "PD-L1"[Title/Abstract]OR "Programmed Death 1"[Title/Abstract] OR "Programmed Cell Death 1 Receptor"[Title/Abstract] OR "Programmed Death-Ligand 1"[Title/Abstract] OR "programmed cell death 1 ligand 1 protein"[Title/Abstract] OR "immune checkpoint inhibitor"[Title/Abstract] OR "immune therapy"[Title/Abstract] OR "immunotherapy"[Title/Abstract]))) AND (perioperative[Title/Abstract] OR neoadjuvant[Title/Abstract] OR adjuvant[Title/Abstract])) AND (((((("nonsquamous"[Title/Abstract]) AND "lung cancer"[Title/Abstract]) AND (((((NSCLC[Title/Abstract]) OR "Non Small Cell"[Title/Abstract]) OR "Non-Small- Cell"[Title/Abstract]) OR "Non-Small Cell"[Title/Abstract]))) OR "Carcinoma, Non-Small-Cell Lung"[Mesh]) AND (("clinical trials as topic "[MeSH Terms] OR "Randomized clinical trial"[Title/Abstract] OR "phase"[Title/Abstract])))

Embase:243 results

(‘pembrolizumab’/exp OR ‘lambrolizumab’:ab,ti OR ‘Keytruda’:ab,ti OR ‘MK-3475’:ab,ti OR ‘nivolumab’/exp OR ‘MDX-1106’:ab,ti OR ‘ONO-4538’:ab,ti OR ‘BMS-936558’:ab,ti OR ‘Opdivo’:ab,ti OR ‘atezolizumab’/exp OR ‘MPDL3280A’:ab,ti OR ‘Tecentriq’:ab,ti OR ‘RG7446’:ab,ti OR ‘RG-7446’:ab,ti OR ‘Durvalumab’:ab,ti OR ‘Imfinzi’:ab,ti OR ‘MEDI4736’:ab,ti OR ‘Camrelizumab’/exp OR ‘SHR-1210’:ab,ti OR ‘Tislelizumab’/exp OR ‘Sintilimab’/exp OR ‘IBI 308’:ab,ti OR ‘anti-PDL1’:ab,ti OR ‘anti-PD1’:ab,ti OR ‘PD-1’:ab,ti OR ‘PD-L1’ab,ti OR ‘Programmed Death 1’ab,ti OR ‘Programmed Cell Death 1 Receptor’:ab,ti OR ‘Programmed Death-Ligand 1’:ab,ti OR ‘programmed cell death 1 ligand 1 protein’:ab,ti OR ‘immune checkpoint inhibitor’:ab,ti OR ‘immune therapy’:ab,ti OR ‘immunotherapy’:ab,ti) OR (‘perioperative’:ab,ti OR ‘neoadjuvant’:ab,ti OR ‘adjuvant’:ab,ti) AND ('non small cell lung cancer'/exp OR 'lung cancer':ab,ti OR 'NSCLC':ab,ti OR 'non small cell':ab,ti OR 'non-small-cell':ab,ti OR 'non-small cell':ab,ti) AND (‘randomized controlled trial’/exp)

Cochrane:590 results

#1 MeSH descriptor: [Carcinoma, Non-Small-Cell Lung] explode all trees

#2 (lung OR pumlon*):ti,ab,kw

#3 (cancer or carcinoma or neoplas*):ti,ab,kw

#4 #2 AND #3

#5 #1 OR #4

© 2024 Zhou Y et al. *JAMA Network Open.*

#6 (perioperative OR neoadjuvant OR adjuvant):ti,ab,kw

#7 (pembrolizumab OR lambrolizumab OR Keytruda OR MK-3475 OR nivolumab OR MDX-1106 OR ONO-4538 OR BMS-936558 OR Opdivo OR atezolizumab OR MPDL3280A OR Tecentriq OR RG7446 OR RG-7446 OR Durvalumab OR Imfinzi OR MEDI4736 OR Camrelizumab OR SHR-1210 OR Tislelizumab OR Sintilimab OR 'IBI 308' OR 'anti-PDL1' OR 'anti-PD1' OR 'PD-1' OR PD-L1 OR 'Programmed Death 1' OR ‘Programmed Cell Death 1 Receptor' OR 'Programmed Death-Ligand 1' OR ‘programmed cell death 1 ligand 1 protein' OR ‘immune checkpoint inhibitor’ OR 'immune therapy’ OR immunotherapy): ti, ab, kw

#8 #5 AND #6 AND #7

© 2024 Zhou Y et al. *JAMA Network Open.*

eTable 1. Quality Assessment by Cochrane Collaboration's Tool

Trial	Sequence generation	Allocation concealment	Blinding	Incomplete outcome data	Selective reporting	Other source of bias
NADIM II	Adequate	Not clear	Adequate	Adequate[a]	Adequate	
KEYNOTE-671	Adequate	Adequate (Central allocation)	Adequate	Adequate[a]	Adequate	
CheckMate 816	Adequate	Adequate (Central allocation)	Adequate	Adequate[a]	Adequate	
AEGEAN	Adequate	Not clear	Adequate	Adequate[a]	Adequate	
Neotorch	Adequate	Not clear	Adequate	Adequate[a]	Adequate	Data from conferences

[a] data concerning event-free survival were adequate, while data of overall survival (OS) has yet to mature in the trial.

© 2024 Zhou Y et al. *JAMA Network Open.*

eTable 2. Additional Characteristics of Patients and Outcomes of Included Trials

**Trial name**	**CheckMate 816**		**KEYNOTE-671**		**Neotorch**		**AEGEAN**		**NADIM II**	
**Arm**	nivo+chemo (n=179)	chemo (n=179)	pembro+chemo (n=397)	chemo (n=400)	tori+chemo (n=202)	chemo (n=202)	durva+chemo (n=366)	chemo (n=374)	nivo+chemo (n=57)	chemo (n=29)
**Median Age** - yr	64	65	63	64	62	61	65	65	65	63
**Region - no. (%)**										
Non-asia	94 (52.5)	87 (48.6)	273(68.8)	275 (68.8)	0 (0.0)	0 (0.0)	224 (61.2)	211 (56.4)	NR	NR
Asia	85 (47.5)	92 (51.4)	124 (31.2)	125 (31.2)	202 (100.0)	202(100.0)	142 (38.8)	163 (43.6)	NR	NR
**Histology - no. (%)**										
Squamous	87 (48.6)	95 (53.1)	171 (43.1)	173 (43.2)	157 (77.7)	157 (77.7)	170 (46.2)	191 (51.1)	21 (37.0)	14 (48.0)
Nonsquamous	92 (51.4)	84 (46.9)	226 (56.9)	227 (56.8)	45 (22.3)	45 (22.3)	196 (53.6)	179 (47.9)	36 (63.0)	15 (52.0)
**PD-L1 status - no. (%)**										
<1%	78 (43.6)	77 (43.0)	138 (34.8)	151 (37.8)	69 (34.2)	70 (34.7)	122 (33.3)	125 (33.4)	NR	NR
≥1%	89 (49.7)	89 (49.7)	NR	NR	133 (65.8)	132 (65.3)	NR	NR	NR	NR
1–49%	51 (28.5)	47 (26.3)	127 (32.0)	115 (28.8)	NR	NR	135 (36.9)	142 (38.0)	NR	NR
≥50%	38 (21.2)	42 (23.5)	132 (33.2)	134 (33.5)	NR	NR	109 (29.8)	107 (28.6)	NR	NR
**pCR- no. (%)**	43 (24.0)	4 (2.2)	72 (18.1)	16 (4.0)	50 (24.8)	2 (1.0)	63 (17.2)	16 (4.3)	21 (37.0)	2 (7.0)
**MPR- no. (%)**	66 (36.9)	16 (8.9)	120 (30.2)	44 (11.0)	98 (48.5)	17 (8.4)	122 (33.3)	46 (12.3)	30 (53.0)	4 (14.0)
**Resectio§ - no. (%)**										
R0	124 (83.2)	105 (77.8)	299 (92.0)	267 (84.2)	159 (95.8)	137 (92.6)	269 (94.7)	262 (91.3)	50 (94.0)	17 (85.0)
R1	16 (10.7)	21 (15.6)	17 (5.2)	31 (9.8)	NR	NR	NR	NR	1 (1.9)	2 (10.0)
R2	5 (3.4)	4 (3.0)	5 (1.2)	5 (1.3)	NR	NR	NR	NR	0 (0.0)	1 (5.0)
Rx (unknown)	4 (2.7)	5 (3.7)	6 (1.5)	30 (7.4)	NR	NR	NR	NR	2 (3.8)	0 (0.0)
**Treatment - no. (%)**										
Neoadjuvant†	165 (93.8)	149 (84.7)	295 (74.5)	287 (74.4)	176 (87.1)	185 (91.6)	310 (84.7)	326 (87.2)	NR	NR
Adjuvant‡	35 (19.9)	56 (31.8)	290 (73.2)	267 (66.9)	144 (71.3)	131 (64.9)	241 (65.8)	237 (63.4)	NR	NR
**EGFR mutation- no. (%)**	0 (0.0)	0 (0.0)	14 (3.5)	19 (4.8)	0 (0.0)	0 (0.0)	0 (0.0)	0 (0.0)	0 (0.0)	0 (0.0)
**ALK translocation-no. (%)**	0 (0.0)	0 (0.0)	12 (3.0)	9 (2.2)	0 (0.0)	0 (0.0)	0 (0.0)	0 (0.0)	0 (0.0)	0 (0.0)
**Tumor stage - no. (%)**										
T1	NR	NR	55 (13.9)	61 (15.2)	NR	NR	NR	NR	NR	NR
T2	NR	NR	106 (26.7)	126 (31.5)	NR	NR	NR	NR	NR	NR
T3	NR	NR	121 (30.5)	109 (27.2)	NR	NR	NR	NR	NR	NR
T4	NR	NR	115 (29.0)	104 (26.0)	NR	NR	NR	NR	NR	NR

© 2024 Zhou Y et al. *JAMA Network Open.*

**Node stage - no. (%)**										
N0	NR	NR	148 (37.3)	142 (35.5)	17 (8.4)	18 (8.9)	NR	NR	6 (11.0)	9 (31.0)
N1	NR	NR	81 (20.4)	71 (17.8)	46 (22.8)	39 (19.3)	NR	NR	10 (18.0)	4 (14.0)
N2	NR	NR	168 (42.3)	187 (46.8)	138 (68.3)	145 (71.8)	NR	NR	41 (72.0)	16 (55.0)

†Patients received completed neoadjuvant treatment.

‡Patients received any adjuvant treatment.

§Denominator based on patients with definitive surgery. (N=149 in the nivolumab plus chemotherapy group, N=135 in the chemotherapy group in CheckMate 816; N=325 in the pembrolizumab plus chemotherapy group, N=317 in the chemotherapy group in KEYNOTE-671; N=161 in the toripalimab plus chemotherapy group, N=148 in the chemotherapy group in Neotorch; N=284 in the durvalumab plus chemotherapy group, N=287 in the chemotherapy group in AEGEAN)

Abbreviations: pCR, pathological complete response; PD-L1, programmed death ligand 1; MPR, major pathological response; N, number; chemo, chemotherapy; nivo, nivolumab; durva, durvalumab; tori, toripalimab; pembro, pembrolizumab; yr, year; NR, not report.

© 2024 Zhou Y et al. *JAMA Network Open.*

eFigure 1. Forest Plot of Hazard Ratios in Subgroup-Analyses Comparing EFS in Patients Who Received Neoadjuvant-Adjuvant Anti-PD-(L)1 Therapy Versus Chemotherapy Alone in Completely Resected NSCLC

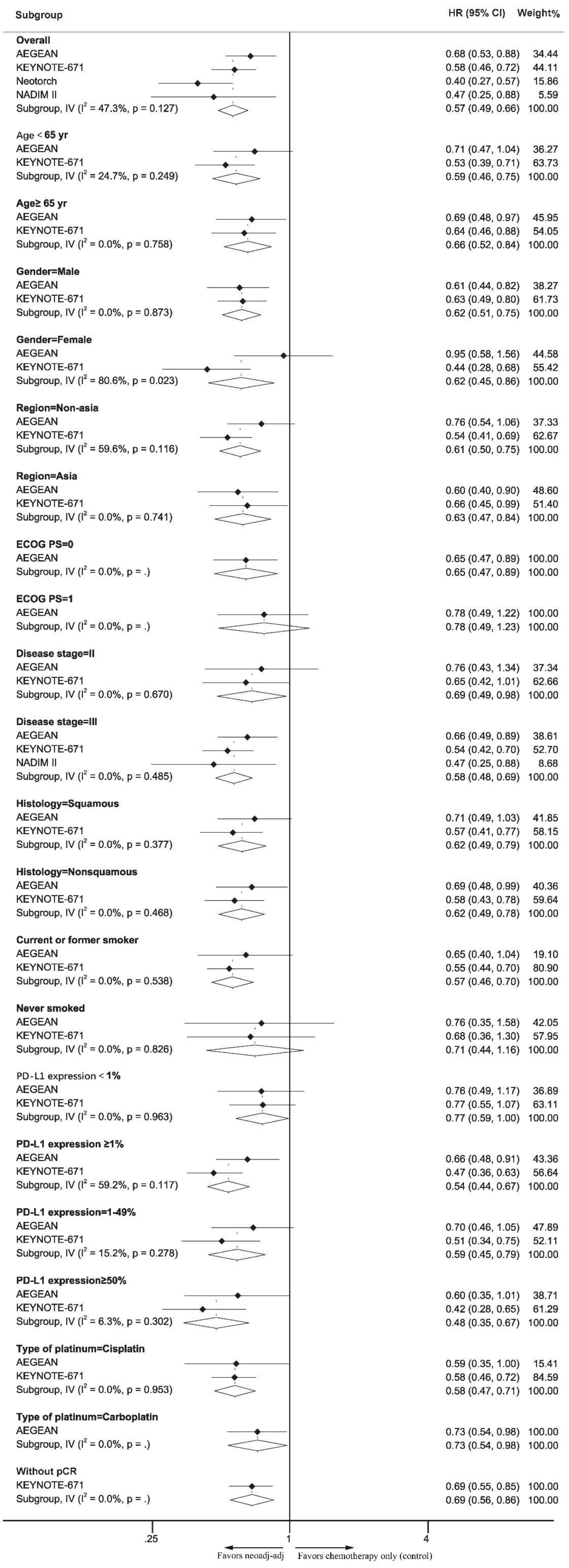

The horizontal line crossing the dot represents the 95%CI of the pooled hazard ratio in each subgroup-analysis. I^2 *(P)* shows the heterogeneity in each subgroup meta-analysis.Abbreviations: NSCLC, non-small cell lung cancer; neoadj-adj, neoadjuvant immunotherapy plus chemotherapy, followed by adjuvant immunotherapy; EFS, event-free survival; HR, hazard ratio; yr, year; ECOG: Eastern Cooperative Oncology Group; PD-L1: programmed cell death-ligand 1; pCR, pathological complete response

© 2024 Zhou Y et al. *JAMA Network Open.*

eFigure 2. Funnel Plot Comparing Hazard Ratios Between Neoadjuvant- Adjuvant Anti-PD-(L)1 Therapy Over Chemotherapy Alone for EFS

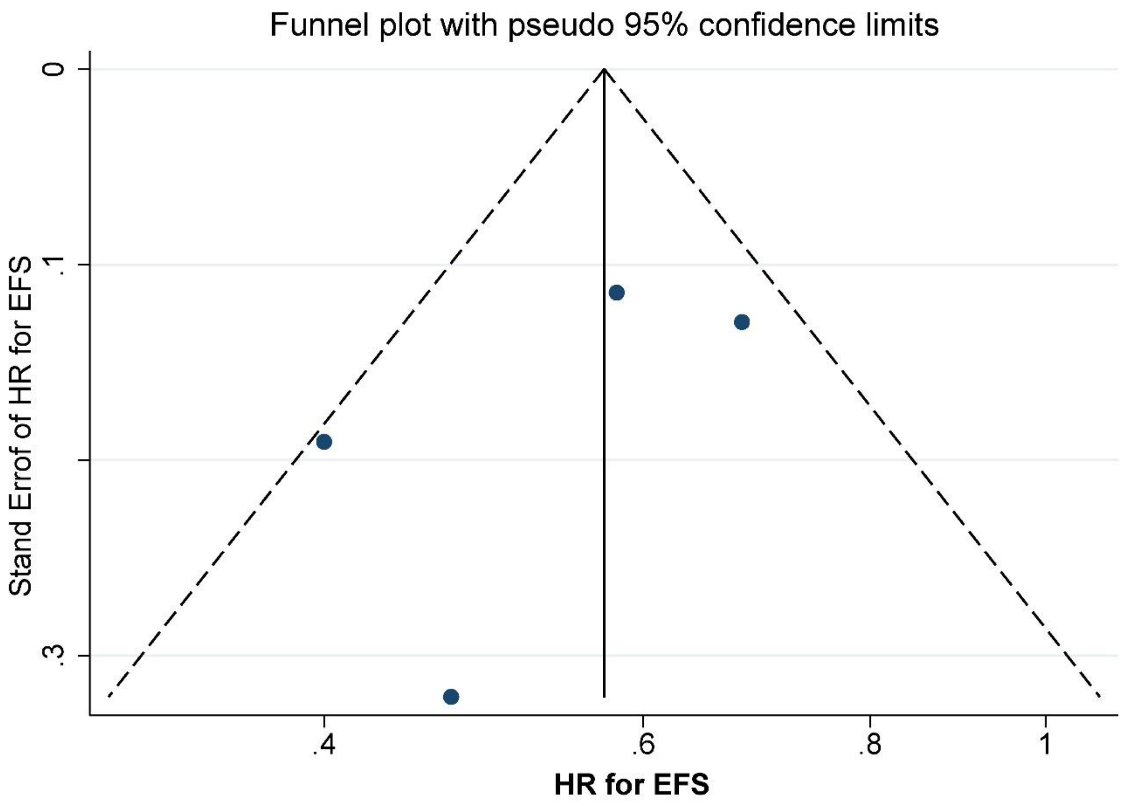

Each study's effect estimate plotted against its standard error. The outer dashed lines represent the confidence interval boundary within which 95% of studies are expected to lie in the absence of bias or heterogeneity. The solid vertical line represents the summary treatment effect. Abbreviations: HR, hazard ratio; EFS, event-free survival; PD-1: program cell death 1; PD-L1: programmed cell death ligand 1.

© 2024 Zhou Y et al. *JAMA Network Open.*

eFigure 3. Forest Plot of Hazard Ratios Comparing OS in Patients Who Received Neoadjuvant- Adjuvant Anti-PD-(L)1 Therapy Versus Chemotherapy Alone in Completely Resected NSCLC

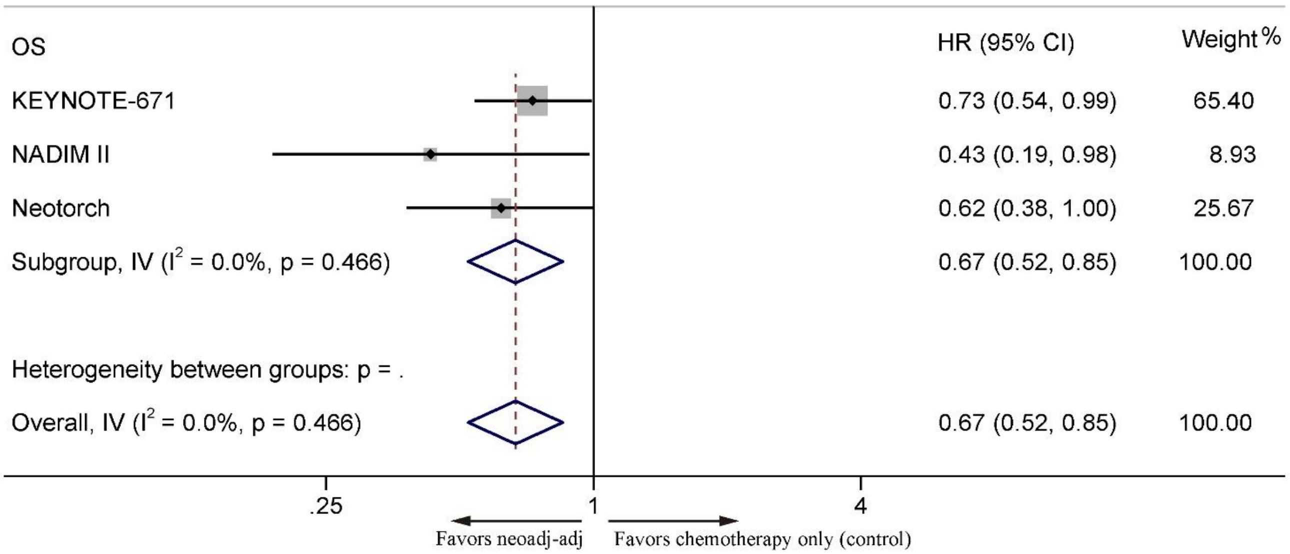

The horizontal line crossing the dot represents the 95%CI of the pooled hazard ratio in the meta-analysis. I^2 *(P)* shows the heterogeneity in the meta-analysis. Abbreviations: NSCLC, non-small cell lung cancer; neoadj-adj,neoadjuvant immunotherapy plus chemotherapy, followed by adjuvant immunotherapy; OS, overall survival; HR, hazard ratio.

© 2024 Zhou Y et al. *JAMA Network Open.*

eFigure 4. Forest Plot of Risk Ratios Comparing TRAES in Patients Who Received Neoadjuvant-Adjuvant Anti-PD-(L)1 Therapy Versus Chemotherapy Alone in Completely Resected NSCLC

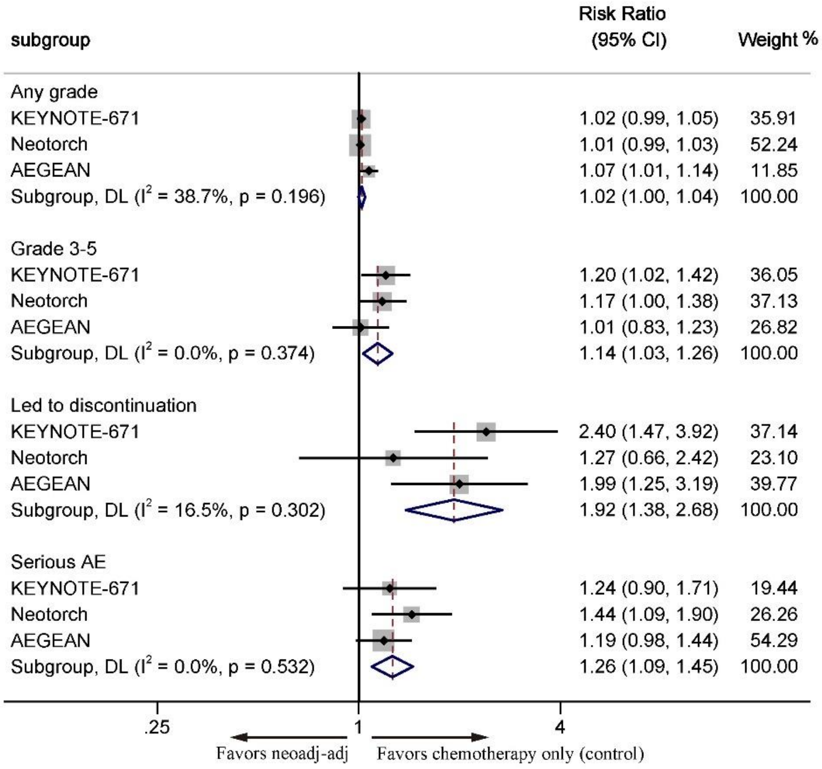

The horizontal line crossing the dot represents the 95%CI of the pooled risk ratio in each subgroup-analysis. I^2 *(P)* shows the heterogeneity in the meta-analysis. Abbreviations: NSCLC, non-small cell lung cancer; neoadj-adj, neoadjuvant immunotherapy plus chemotherapy, followed by adjuvant immunotherapy; TRAEs, treatment-related adverse events; AEs, adverse events; PD-1: program cell death 1; PD-L1: programmed cell death- ligand 1.

© 2024 Zhou Y et al. *JAMA Network Open.*

eFigure 5. Forest Plot of Risk Ratios Comparing TRAES in Patients Who Received Neoadjuvant-Only Anti-PD-(L)1 Therapy Versus Chemotherapy Alone in Completely Resected NSCLC

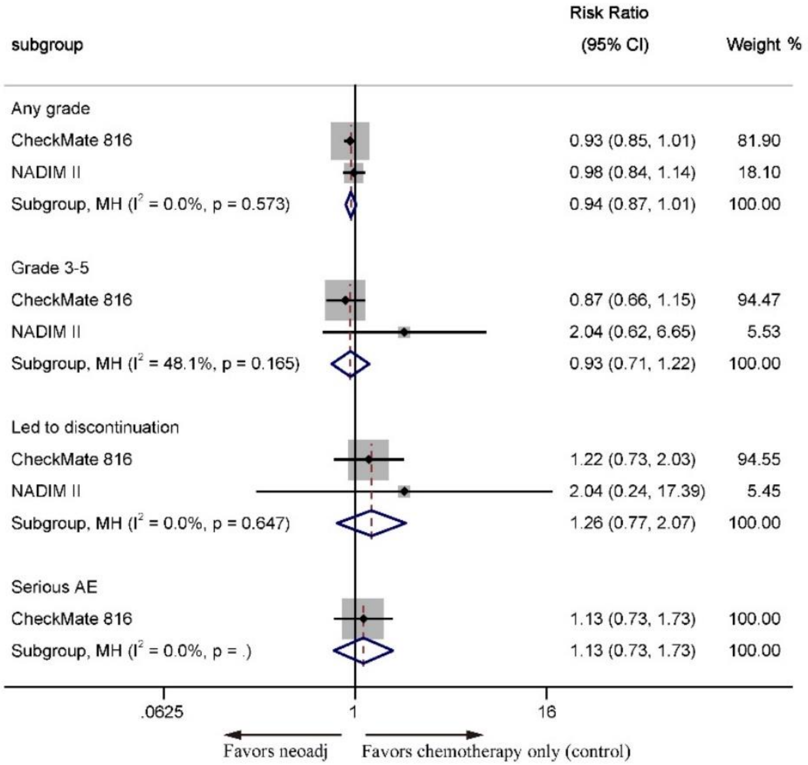

The horizontal line crossing the dot represents the 95%CI of the pooled risk ratio in each subgroup-analysis. I^2 *(P)* shows the heterogeneity in the meta-analysis. Abbreviations: NSCLC, non-small cell lung cancer; neoadj, neoadjuvant immunotherapy plus chemotherapy; TRAEs, treatment-related adverse events; AEs, adverse events; PD-1: program cell death 1; PD-L1: programmed cell death-ligand 1.

© 2024 Zhou Y et al. *JAMA Network Open.*
