# Supplementary material for: Neoadjuvant-Adjuvant vs Neoadjuvant-Only PD-1 and PD-L1 Inhibitors for Patients With Resectable NSCLC: An Indirect Meta-Analysis
Source: JAMA Netw Open. 2024 Mar 7;7(3):e241285. doi: 10.1001/jamanetworkopen.2024.1285 (PMC10921251; doi:10.1001/jamanetworkopen.2024.1285)
Supplement: Supplement 2. — Data Sharing Statement [file jamanetwopen-e241285-s002.pdf]

Data Sharing Statement

Zhou. Neoadjuvant-Adjuvant vs Neoadjuvant-Only PD-1 and PD-L1 Inhibitors for Patients With Resectable NSCLC. *JAMA Netw Open*. Published March 07, 2024.
doi:10.1001/jamanetworkopen.2024.1285

Data
Data available: No

Additional Information
Explanation for why data not available: All data generated or analysed during this study are included in the published article or conference.
